# Philadelphia Polyclinic Medical Society

**Published:** 1891-11

**Authors:** Thomas J. Mays


### SWEET OIL IN THE TREATMENT OF GALL-STONES.

### A COLLECTIVE INVESTIGATION BY THE THERAPEUTIC SECTION OF THE PHILADELPHIA POLYCLINIC MEDICAL SOCIETY.

By THOMAS J. MAYS, M. D

The subject of the action of sweet oil in the treatment of biliary colic and catarrh of the hepatic passages has recently been warmly discussed. There are many who regard this agent as being very much overrated, while many others believe that it has a very beneficial influence on this disease. In view of the divided opinions on, and the importance of, this matter, the Therapeutic Section of the Philadelphia Polyclinic Medical Society has, as a part of its scientific work, undertaken a special collective investigation concerning the clinical value of this drug in gall-stone colic. With this end in view, the undersigned committee was appointed, and directed to send a number of circulars to the members of the profession, of which the following is a copy :

Sex and age of patient ? Seat of pain ? Jaundice ? Previous attacks ? Did you test any other remedy, and with what results ? Result of treatment with olive oil. Remarks.

To these circulars nineteen replies were received, and thirty-seven cases of gall-stone colic treated with olive oil were reported. To these members of the profession the warmest thanks of this committee are due for the promptitude with which they responded. Additionally the committee imposed the task upon itself to collect, as far as possible, all the previously reported cases of biliary colic which were treated according to this method, and succeeded in gathering records of seventeen cases, making altogether a list of fifty-four cases, a condensed history of which is presented in the following table :

1. Read September 9, 1891.

### Table of Cases.

| No. | Sex and age. | Seat of pain. | Jaundice. | Previous attacks. | Use of other remedies, and results obtained. | Results obtained from the use of sweet oil | Remarks. | Name and address of observer. |
|---|---|---|---|---|---|---|---|---|
| 1 | F. 40 | Right hypochondrium. | Yes. | Three or four. | None. | Six ounces taken in 3 hours. Relief in twenty-four hours. | No recurrence for more than 3 years, up to time of report. | H. T. Bahnson, Salem, N. C. |
| 2 | M. 50 | Right hypochondrium. | Yes. | A great many. | Antipyrin hypodermically, with temporary relief. | One pint taken in two hours; complete relief. | No return for more than 2 years. | H. T. Bahnson, Salem, N. C. |
| 3 | F. 65 | Right hypochondrium. | Yes. | Five or six. | None. | Half a pint taken in four hours; relief in 12 hours | No return for more than 3 years. In two other cases the single large dose produced relief, but failed to prevent a recurrence of attacks. | H. T. Bahnson, Salem, N. C. |
| 4 | M. 46 | Right hypochondrium. | Yes. | Three. | Chelidonium and dioscorea gave some relief. | Quantity of oil given not stated Remained well so long as he took it. | Used it in other cases of biliary troubles, and with good success. | G. R. Fortiner, Camden, N. J. |
| 5 | M. 34 | Right hypochondrium. | Yes. | No. | Chelidonium without relief. | Quantity of oil given not stated. Administered it for 10 days, when patient died. | *Post-mortem* investigation showed complete adhesive obstruction of bile ducts. Patient received a blow in hepatic region some time before. | G. R. Fortiner, Camden, N. J. |
| 6 | F. 28 | Gastric region. | Yes. | Eight or ten. | Sodium phosphate without benefit. | One pint at a single dose. Complete relief. | No recurrence within a year. | J. J. Cox, High Point, N. C. |
| 7 | F. 49 | Epigastrium. | Yes. | Six. | Sodium phosphate with some benefit. | Took the oil for 4 weeks. Dose not stated. | No recurrence; general condition much improved. | G. H. Franklin, Hightstown, N. J. |
| 8 | M. 67 | Epigastrium. | Yes. | Twelve | Sodium phosphate, after which a severe attack became less frequent. | Continued oil for 6 weeks. Dose not given. | No recurrence; improvement after oil surprising. | G. H. Franklin, Hightstown, N. J. |
| 9 | M. 45 | Right hypochondrium. | No. | One a week for 3 months | None. | Took the oil for 4 weeks. Dose not given. | One light attack since he began the oil. | G. H. Franklin, Hightstown, N. J. |
| 10 | M. 31 | Right hypochondrium. | Yes. | Once every 3 weeks during 14 years | Morphine and anesthetics; temporary abatement. | Dose of oil not stated. Free from attacks for 11 months. | Her previous sufferings were intense, requiring large doses of narcotics. | A. B. Gloninger, Lebanon, Pa. |
| 11 | F. 35 | Right hypochondrium. | Yes. | Uncertain. | None. | Dessertspoonful of oil every 3 hours. Relieved after second dose. | History of malaria, liver and spleen enlarged. | E. Lawney, Denver, Col. |
| 12 | M. 10 | Right hypochondrium. | Yes. | One 5 years before. | None. | Dessertspoonful of oil. Pain relieved at once. | Also gave ammon. chloride, gr. iij., and calomel 1-32 gr. t. i. d | E. Lawney, Denver, Col. |
| 13 | F. 51 | Hepatic and gastric region. | Yes. | No. | Yes; nature of same not mentioned. | Daily for 2 days gave 8 ounces of oil; no relief. | Patient died. | E. P. Bernardy, Philadelphia. |
| 14 | F. 72 | Region of gall-bladder. | Yes. | No. | Yes; nature of same not mentioned; no benefit. | Nine ounces of oil for 10 days without positive improvement. | Oil caused numerous alvine discharges, lightened the color of skin, and seemed to reduce size of gall bladder. | E. P. Bernardy, Philadelphia. |

TABLE OF CASES.—*Continued.*

| No. | Sex and age. | Seat of pain. | Jaundice. | Previous attacks. | Use of other remedies, and results obtained. | Results obtained from the use of sweet oil. | Remarks. | Name and address of observer. |
|---|---|---|---|---|---|---|---|---|
| 15 | M. 40 | Right hypochondrium. | Yes. | Several but none for 5 years. | Dioscorea, morphine, and atropine, with some relief. | Dessertspoonful every half hour with the most marked relief. | Regulated diet, and gave sodium phosphate, etc. | Theo. G. Davis, Bridgeton, N. J. |
| 16 | F. 30 | Over abdomen. | Yes. | One, possibly two | Calomel, sod. bi-carb. and morphine, slight relief. | Dessertspoonful of oil every 3 hours; complete relief after second dose. | Stools contained concretions. | E. H. Bidwell, Vinland, N. J. |
| 17 | F. 55 | Right hypogastrium. | Yes. | Yes. | Morphine and atropine hypodermically, with what results not stated. | Gave 6 ounces of oil, and relief came in an hour. Following day slight attack; 10 ounces. No recurrence. | The intense vomiting from which she suffered ceased after the oil was taken. | Ch. Pottberg, Philadelphia. |
| 18 | M. 40 | Right hypochondrium. | Yes. | Several | Silver nitrate, regulation of diet and water with good results. | Dose of oil not stated. Negative results. | Examination of feces after oil showed contents of soapy concretions. | J. Daland. |
| 19 | M. 50 | Right side of abdomen | Yes. | At least two. | Chloroform inhalation and sodium bromide; did not obtain decided relief until oil was taken. | Relieved after taking 3 doses (size not stated) of oil. | The oil appeared to relieve him, but he may also have been helped by the chloroform. | |
| 20 | M. 46 | Over gall-bladder on pressure. | Yes. | Two. | Not stated. | Ten hours after taking 1 quart of oil in divided doses, 2 large gall-stones discharged in the stools. Steady improvement. | Bowels had not been moved for 4 days before the oil was taken. Singultus existed for 12 hours before bowels moved. | A. F. Magruder, U. S. N., Wash., D. C. |
| 21 | M. 52 | Over gall-bladder. | Yes. | For years at intervals of from 4 to 6 months | Nature of, not stated; temporary relief. | Half-ounce doses of oil every 5 hours for about a month before report was made. | Too early to judge the effect of oil. General health better than for two months. | J. D. Dewitt. |
| 22 | F. 40 | Right hypochondrium. | Yes. | One about 2 months before. | Morphine, quinine, atropine, calomel, etc. Not the prompt relief obtained with the oil. | Dessertspoonful of oil every 4 hours. Improvement at once. | No recurrence so far as known. Gall-bladder diminished in size. | Thos. J. Mays, Philadelphia. |
| 23 | F. 45 | Right hypochondrium. | Yes. | Periodically for a number of years | Morphine and atropine hypodermically gave slight temporary relief. | Dessertspoonful of oil every 3 hours, with decided relief. | No attack since so far as known. | Thos. J. Mays, Philadelphia. |
| 24 | M. 20 | Not known. | Yes. | During previous 3 months | No. | Dessertspoonful of oil 3 times a day with prompt relief. | No return so far as can be learned. | Thos. J. Mays, Philadelphia. |
| 25 | M. 27 | Right hypochondrium. | Yes. | Periodical during previous year. | Not by observer. | Dessertspoonful of oil 4 times a day with gradual relief. | No recurrence. | Thos. J. Mays, Philadelphia. |
| 26 | M. 67 | Right hypochondrium. | No. | No. | None used. | Dose of oil not given. Complete relief. | Hard concretions like gall-stones passed freely for 10 days after taking the oil. | C. R. Early, Ridgway, Pa. |
| 27 | F. 35 | Right hypochondrium. | Yes. | Very frequent. | Morphine, mercury, and potassium iodide; good results. | Dose of oil not stated. Good results. | Perfectly well in 3 or 4 weeks after taking the oil. | C. R. Early, Ridgway, Pa. |

TABLE OF CASES.—*Continued.*

| No. | Sex and age. | Seat of pain. | Jaundice. | Previous attacks. | Use of other remedies, and results obtained. | Results obtained from the use of sweet oil. | Remarks. | Name and address of observer. |
|---|---|---|---|---|---|---|---|---|
| 28 | M. 23 | Righthypochondrium. | Yes. | Yes. | Potassium chlorate, sodium bi-carbonate, and ipecac; good results. | Dose of oil not stated. Cured in 2 weeks. | No recurrence for 3 years. | C. R. Early, Ridgway, Pa. |
| 29 | F. 50 | Righthypochondrium. | Yes. | Yes. | None by observer, but by other physicians. | Dose of oil not stated. Found relief in 2 days. | No recurrence; treated 40 years ago. Treated many similar cases during this time. | C. R. Early, Ridgway, Pa. |
| 30 | F. 54 | Righthypochondrium. | Yes. | Quite a number. | Of everything else he tested, chloroform seemed to give the best results; relief temporary. | Six ounces of oil in 2 equally divided doses, half an hour apart. Gave oil at 3 different times. No symptoms for 2 years. | Passed a large number of calculi. Enforces a rigid dietary in all these cases. Allows no sugar starchy or fatty foods. | D. P. Boyer, Philadelphia. |
| 31 | M. 60 | Righthypochondrium. | Yes. | A number during 3 previous years. | Sodium phosphate, chloroform, morphine, and succinic acid; no satisfactory results. | Same dose as in previous case. Relieved 2 attacks 2 months apart. | No recurrence since second attack, which occurred a year and a half ago. | D. P. Boyer, Philadelphia. |
| 32 | F. 22 | Righthypochondrium. | Yes. | Two. | Sodium phosphate, morphine, mineral water, etc., seemed to relieve first, but not second attack. | Only received the oil for 2 days, when she was entirely relieved. Passed a number of calculi. | Treated about 10 cases with the oil, and in all there was either a cure or benefit. | D. P. Boyer, Philadelphia. |
| 33 | M. 42 | Righthypochondrium and in epigastrium. | Yes. | None. | Tested numerous chologogues without benefit. | Six ounces of oil at night, followed next morning with laxative; relief. | Discharge of biliary calculi. No recurrence. | Ed.R.Mayer, Wilkesbarre, Pa. |
| 34 | F. 40 | Righthypochondrium. | Yes. | About 2 a year for 15 years. | Not by observer. | Six ounces of the oil gave prompt relief. This was the last attack the patient had. | Insists on a rigid dietary. Chologogues,Carlsbad waters, etc., as preventatives. | Ed.R.Mayer, Wilkesbarre, Pa. |
| 35 | M. 58 | Righthypochondrium and right shoulder. | Yes. | No. | Not by observer. | Same dose of oil in the evening and purgative in the morning. Complete relief after 4th dose. No recurrence. | Gall-bladder was so distended with calculi that it could easily be mapped out. Treated about 35 cases of gall-stones during last 14 years with olive oil, and in every instance the severity of the attack was mitigated by the first and entirely relieved by third or fourth dose. | Ed.R.Mayer, Wilkesbarre, Pa. |
| 36 | F. 42 | Righthypochondrium and back. | Yes. | Suffered for 10 years off and on. | Sodium phosphate gave temporary but no permanent relief like the oil. | Tablespoonful of oil every 3 hours for about 1 month; after an interval gave it again, but less frequently. Pain ceased at once after oil was administered. | No recurrence: passed a calculus in feces weighing 40 grs. | J. S. Baer, Camden, N. J. |

TABLE OF CASES.—*Continued.*

| No. | Sex and age. | Seat of pain. | Jaundice. | Previous attacks. | Use of other remedies, and results obtained. | Results obtained from the use of sweet oil. | Remarks. | Name and address of observer. |
|---|---|---|---|---|---|---|---|---|
| 37 | M. 68 | Epigastrium and right hypochondrium. | Yes. | Two. | Opium and ether only gave temporary relief. | Dessertspoonful doses of the oil gave prompt and decided relief. | No recurrence. | H. C. Bloom, Philadelphia. |
| 38 | F. 42 | Region of gall-bladder. | Yes. | Yes. | Morphine hypodermically and by the mouth; ether inhalation and hot poultices, without relief. | Half an hour after swallowing from half to three-fourths of a pint of oil the pain ceased abruptly. | First 3 passages after oil contained 2 gall-stones; has had 2 slight attacks, since which did not require medical interference. | D.D.Stewart, Med News, Nov. 23, 1889. |
| 39 | F. 45 | Right hypochondrium. | Yes. | Yes, for 12 years. | Morphine and atropine hypodermically; hot water, etc., relief for 2 hours when pain returned as severely as before; morphine gave no relief. | Forty-five minutes after being able to retain 5 ounces of *cotton-seed* oil, pain diminished, and ceased 3 hours later. | Subsequent attack also relieved by the oil. Fifth day after last attack passed a calculus as large as a beech nut. | D.D.Stewart, Med. News, Nov. 23, 1889. |
| 40 | M. elderly. | ...... | Yes. | For some years. | Usual remedies, without relief. | Six ounces of oil at bedtime, followed by castor oil next morning. Passage of gall-stones. Relief. | In 2 days another paroxysm of pain was threatened. Ordered oil 2 following nights. Saw her four months after. No recurrence. | R. Kennedy, Kingston, Can., Lancet, 1890, vol. ii., p. 456. |
| 41 | F. adult | ...... | ... | For years. | Not stated. | Full doses of oil for 2 consecutive days. No return. | Passed a large number of calculi. Relieved two other cases of gall-stone colic with the oil. | R. Kennedy, Kingston, Can., Lancet, 1890, vol. ii., p. 456. |
| 42 | F. 40 | ...... | ... | Yes. | Treated by other physicians without relief. | Eight ounces of oil at bedtime, and following morning after last a dose of castor oil. Relief. | Evacuated several small gall-stones. | Dr. Gay, Buffalo, Buff. Med. and Surg. Journ., vol. vi., p. 214, 1866–67. |
| 43 | M. adult | ...... | ... | Third attack. | Morphine hypodermically to allay pain. | Eight ounces of oil night and morning, followed by a dose of podophyllin. Relief. | Says that olive oil is as much a specific in gall-stone colic as sulphur is in scabies. | Dr. Gay, Buffalo, Buff. Med. and Surg. Journ., vol. vi., p. 214, 1866–67. |
| 44 | F. adult | Pain in epigastrium. | Yes. | Yes. | Was treated for scirrhus of the liver by other physicians. Received 60 drops of McMunn's elixir of opium, with only temporary benefit. | Eight ounces of oil for 2 consecutive nights. Evacuated calculi. Relief. | Success in this case led him to use it in another, which is not fully described. | Ira Hatch, Chicago, Ill., Chicago Med. Examiner, 1867, vol. viii., p. 469. |
| 45 | ... adult | ...... | ... | At least one attack before. | One-eighth gr doses of calomel; bowels moved; no relief; patient vomited everything. | Two pints of oil in divided doses. Relief. The oil was the only thing which would remain in stomach. | An operation had been suggested, but with the improvement it was abandoned. | F. W. Langdon, Cin. Lancet-Clinic, 1890, p. 191. |

TABLE OF CASES.—*Continued.*

| No. | Sex and age. | Seat of pain. | Jaundice. | Previous attacks. | Use of other remedies, and results obtained. | Results obtained from the use of sweet oil. | Remarks. | Name and address of observer. |
|---|---|---|---|---|---|---|---|---|
| 46 | F. 36 | Liver enlarged and sensitive; gall-bladder enlarged. | ... | Almost daily for 5 years. | Obtained no relief from other treatment. | Large doses of oil for 2 weeks. Relief. | Free from attacks for 18 months, up to the time report is made. Passed hard concretions. | S. Rosenberg, Therapeut. Monatshefte Dec., 1889, vol. iii. |
| 47 | F. 37 | In hepatic region; enlarged liver. | Yes | At least for 5 months attacks occ'rr'd during menstrual periods. | Cathartics and other agents brought no relief. | Twenty-four hours after the first large dose of oil pain disappeared, and in a few days the liver diminished in size. | About 2 months after this attack there was a slight return checked with 1 dose. No recurrence and a general improvement. | S. Rosenberg, Ibid. |
| 48 | F. 38 | ...... | ... | For 9 years; latterly once a week. | Mineral-water cure without benefit. | Large doses of oil relieved her, excepting a slight soreness in region of liver, which was cured with 15 grs. sodium salicylate 3 times a day. | Passed a biliary calculus. Free from pain to time of the report—about one year. | S. Rosenberg, Ibid. |
| 49 | F. 48 | In region of gall-bladder. | Yes. | Several attacks. | ......... | Large doses of oil followed by 2 passages and relief within 24 hours. | Passed large number of concretions. The attack occurred with each menstrual period for some time before she took the oil. | MM. Chauffard et Dupré, Société Médicale des Hôp de Paris, tom. v., 1888. |
| 50 | F. 62 | ...... | Yes. | For 12 years. | ......... | Large dose relieved her for 8 days, but the final results are of a doubtful character. | ..... ...... | MM. Chauffard et Dupré, Ibid. |
| 51 | F. 50 | ...... | Yes. | For a number of years. | ......... | Large doses of oil were followed by local and general improvement. | ............ | MM. Chauffard et Dupré, Ibid. |
| 52 | F. 45 | In hepatic region. | Yes. | At least 3 attacks. | ......... | Large doses of oil followed by beneficial results. | The pain in the hepatic region disappeared, and the colic was cured. | MM. Chauffard et Dupré, Ibid. |
| 53 | F. 58 | In hepatic region. | Yes. | A number of attacks. | ......... | Large doses of oil followed by relief. | Passed a large number of concretions. Pain and swelling in liver disappeared. | MM. Chauffard et Dupré, Ibid. |
| 54 | F. 43 | In hepatic region. | Yes. | Six attacks. | ......... | Large doses of oil followed by relief. | The attacks of biliary colic were associated with nephritic colic, and with the discharge of a urinary calculus, biliary calculi passed at the same time. | MM. Chauffard et Dupré, Ibid. |

An analysis of these fifty-four cases shows that there were about one-third more females than males who suffered from gall-stone colic; that two died, that in three negative results were obtained, and that in fifty, or in ninety-eight per cent., positive relief was afforded. These results make a better showing still, when we consider that one of those who died was suffering from adhesive obstruction of the bile-duct — a disease which no procedure, either medical or surgical, could have remedied. Nor do these figures give us a true estimate of the favorable action of olive oil in this disease; for two of the observers state that they have treated forty other cases of biliary colic without a failure, but of which they had kept no record — making in all a collective return of eighty-nine cases—showing the great value of this drug.

These cases illustrate, then, the positive efficaciousness of sweet oil in the treatment of gall-stone colic, and the question naturally arises, therefore, as to the manner in which this agent acts. Dr. Rosenberg's experiments ("Ueber die Anwendung des Olivenöls bei der Behandlung der Gallensteinkrankheit," *Therapeutische Monatshefte*, December, 1889, S. 542,) demonstrate beyond a doubt that it largely increases the quantity of bile secreted, while at the same time it diminishes its consistency. But how does it accomplish this? Does it stimulate the biliary channels by coming in contact with their openings into the alimentary canal? Or is it decomposed into fatty acids and glycerin through the instrumentality of the pancreatic juice, and does the "glycerin so liberated exert in the duodenum an action similar to that which takes place when it is introduced into the rectum," causing a powerful reflex peristalsis — an ingenious theory suggested by Dr. D. D. Stewart?[1] Or does it act in accordance with the hypothesis formulated by Virchow, who shows from his own experiments (*Therapeutisch Monatshefte*, 1890, S. 86,) that it is absorbed from the alimentary canal, is excreated by the liver, and is thrown into the bowels again through the biliary passages? The last of these theories appears to be most rational, because it explains certain well-known features in its action, and also places it on a level with the action of other cholagogues. We may conceive, then, that the beneficial influence of oil consists not so much in dissolving the biliary concrements, as it does in increasing the biliary excretion, in flushing, and in lubricating and washing out the passages of the liver.

1. A Suggestion as to the Action of Olive or Cotton-Seed Oil in Gall-Stone Colic. By Dr. D. D. Stewart. *Medical News*, November 23, 1889.

Another point of interest in this collection is as to the proper dose of the oil. Are the large doses necessary which were administered to most of the cases in this collection ? It appears not, for eight of the cases (Nos. 11, 12, 15, 16, 22, 23, 24, and 25,) received only dessertspoonful doses every three or four hours, and apparently with the same prompt and positive relief as that which was afforded by doses of from five ounces to one and two pints. If this should be confirmed by further experience, it would be a great practical gain, in view of the fact that a great many persons show a strong aversion to all kinds of oil, especially if they are to be taken in large quantities.

Furthermore, according to the observation of Dr. Stewart (Case 37,) it does not appear to make any difference whether olive or cotton-seed oil is used. Indeed, it is well known that much of the oil which is sold as olive is in reality refined cotton-seed oil ; and Dr. Stewart's observation tends to show that in all probability any bland oil will have the same effect on the disease under consideration.

In conclusion, the committee desires to congratulate the Polyclinic Medical Society on the selection of a subject for collective investigation which has proven so fruitful of practical results as that which is embodied in this report; and expresses the hope that it may continue its good work of testing therapeutic agents in a clinical way. It is true that animal experimentation often points out the path in which the usefulness of a drug lies, but clinical and collective research is, after all, the crucial and final test of all true therapeutic progress.

THOMAS J. MAYS, M. D.,
HOMER C. BLOOM, M. D.,
*Committee.*

DISCUSSION.

DR. WILLIAM S. STEWART: I wish to show the society a stone which was obtained this summer from a lady between seventy and eighty years of age. She had suffered with periodical attacks affecting the bowels and passing off with simple treatment. On the last occasion she suffered excruciating pain in the region of the cecum, and I was sent for. Thinking there might be some inflammatory affection in this region and that possibly abdominal section would be necessary, and finding a mass in the right iliac region, I placed her in the knee-chest position and gave her a very large injection. After as much as possible had been injected, I had her sit on a jar. Not experiencing relief, I continued the injections while she was in this position, using flaxseed tea. This was continued for five or ten minutes. She was then put to bed and another large injection given. I then left her, and shortly afterward she got up and this huge gall-stone was passed. (About the size of a hen's egg.)

Some twenty-five years ago my attention was directed to the method of treatment by olive oil by a lady from California. I had seen her some time before in a debilitated condition from frequent attacks of biliary colic. I inquired the cause of the improvement, and she told me that she went with a friend to see a quack, who at once told her that she had gall-stones, and directed her to take half a pint of sweet oil in the evening, and to rub the right side frequently during the night, telling her that when her bowels were opened she would pass many gall-stones. It happened just as he said, and she had entire relief. Since then I have taken advantage of this hint,

I use the spirits of chloroform in combination with olive oil during the period of attack, and recommend that the oil be continued in doses of two tablespoonfuls before each meal, for a period of several weeks afterward. In this connection I recall to mind an amusing occurrence that happened in the army. A soldier was suddenly taken with biliary colic, and the assistant surgeon, an ignorant man, who had been promoted from the position of hospital steward, was called. He at once prepared a dose of chloroform, and taking it to the patient, said: "Take this; it may do you good or it may kill you; try it."

Dr. John C. DaCosta: My results with olive oil have not been so brilliant nor so quick as those reported in the paper. Opiates and chloroform will relieve the pain, but phosphate of soda does not seem to act as well as reported. Calomel between the attacks acts well; corrosive sublimate still better. One case will illustrate what I have to say. A hysterical woman has had at least five or six attacks in two years. I was disposed to think that all the attacks were not due to gall-stones until she brought me the stones which she had just passed. She was treated with olive oil in half-pint doses. Since then she has had four or five attacks. In the last two she did not consult me until they were over. She takes half a pint to a pint of olive oil at one dose and lies down. In twelve or fourteen hours (not two or three as in some of the reported cases) she is relieved. The oil seems rather to lengthen the interval between the attacks.

Dr. M. Price: The committee deserves great credit for its thorough investigation, but I cannot see the brilliant results referred to.

In my experience I have not found the spasm of biliary colic to last many hours. How olive oil can relieve the pain, unless it is by a lubricating process, I cannot understand. Many stones are of a soft non-irritating nature ; and if the spasm in the gall-duct is relieved, there is no trouble ; the stone passes readily except when the duct is inflamed. Within a month I have seen two post-mortems. In one, a man, there were found two hundred and fifty gall-stones in the bladder. The man had never had an attack. In the other, that of an old lady with cancer of the kidney and a stone in the kidney, the gall-bladder contained five or six ounces of grumous fluid and a number of soft gelatinous stones. She had never had any symptoms referable to the gall-bladder. There is another form of stone in which the treatment could be of no benefit, and that is in those hard ones which we see in cancer, and which in many cases, I think, are the cause of cancer of the liver. Where the stone is large and produces constant irritation there can be no possible benefit from any treatment except tremendous doses of morphia. The stones which I show you are hard and were removed by section.

The history of the cases reported is of this character. You prescribe a remedy to which the patient submits and endures the pain until the spasmodic period of the disease passes. Chloroform and morphia during the continuance of the spasm seems to be the only rational treatment.

In this whole series of cases has there been a post-mortem ? It is very difficult to say when you have a patient suffering with spasmodic pain in the region of the gall-bladder that it is due to gall-stones. Four or five years ago I saw a traveling minstrel who had had attack after attack, and had been treated by the best surgeons in the country for gall-stones. He had received olive oil and many other methods of treatment. I said to him there was no use of doing anything until we found out what was in the gall-bladder. I operated, and after breaking up adhesions reached the gall-bladder and found it perfectly healthy. The patient was completely relieved. His symptoms had simulated gall-stones so much that many surgeons wanted to open him for gall-stones.

In regard to cases of obstruction of the gall-duct.[3] There are cases simulating gall-stones which are not relieved by medical treatment, but which could be relieved without trouble by abdominal section. It has been suggested by one of the best surgeons in this

3. In the doctor's report of the case of stricture of the gall-duct with death, the post mortem found no stone ; in this case it was stricture, and not stone, under treatment.

country to open the abdominal cavity and drain the gall-bladder, then to take a rubber ligature and unite the duodenum or ileum to the gall-bladder, and then with a whipped suture unite the peritoneum of the bowel to the peritoneum of the gall-bladder. In the course of three or four days a fistula forms and then the abdominal opening can be closed with silkworm gut sutures, previously introduced. There is no question that gall-stones of the size that I have shown should be removed. It is my firm belief that gall-stones of this hard, irritating nature may in time produce malignant disease of the gall-bladder or of the gall-duct.

I think with Dr. Mays, that the explanation of the benefit of olive oil, if it exerts any, is to be found in the lubricating and stimulating qualities of the oil, and that it relieves spasm simply by its purgative effect. I cannot see otherwise where the benefit can come in. It has no solvent effect, and it cannot remove a stone whose diameter is ten times that of the gall-duct.

Dr. James B. Walker: In my experience, the question is not so much what will relieve the attack, as what will prevent its recurrence. If one would send out a series of questions in regard to the usefulness of any particular agent in gall-stones, would he not receive replies very similar to those given tonight? Take calomel, or any other agent which acts as a laxative, and it will terminate an attack of gall-stones, if it is a terminable attack. The question is, what will prevent recurrence? So far as this report goes, it seems to give to olive oil a favorable position as an agent for preventing recurrence. This is the most favorable report that I have seen. Still these are not exceptional results. We meet with cases of gall-stones where the attack is never repeated. Unquestionably, in many cases, the recurrences are due to the number of gall-stones. Sometimes, however, the attack terminates without the discharge of the stone, and yet there is no recurrence for months. I have a patient who recently passed a calculus, resembling a mulberry, both in shape and appearance; he passed this after a number of attacks under my observation. The attacks were treated with morphia and calomel, followed by phosphate of sodium and nitro-muriatic acid. After each attack there would be a return to health for a period. In at least ten attacks, the feces were examined without result. At times a detritus was found, looking like a crumbled stone. This was found in the last attack, and it was thought that the stone had crumbled and passed; however, the following day another severe attack occurred, in which blood was passed, and in the center of a clot of blood was found the calculus, as large as the last joint of the index finger, and without facets. This was, unquestionably, the cause of all the trouble.

I believe that any laxative agent which will promote peristalsis and free biliary discharge will relieve the immediate attack if the stone can escape; then some agent to relieve the catarrh of the duodenum and the hepatic catarrh will be required. I have used olive oil in several cases, but my experience has not been satisfactory. The case just referred to received six ounces of olive oil on two occasions without benefit; other patients have received equal doses without success. The best remedy that I have found for old, recurring cases, where there seems to be an impacted stone, and where even cancer has been suspected, from the cachexia, is spirits of chloroform. In two cases, sisters, seen at intervals of five years, this seemed to be the agent that turned the tide of events after repeated efforts with other remedies. I gave it in teaspoonful doses three times a day. The cases immediately changed their character, the jaundice disappeared, and improvement in the general health took place. I believe the chloroform, which is known to increase the liquefaction of the bile, is of value in these cases; this, with phosphate of sodium, or some other sodium salt, or, where the patient is wealthy enough, a visit to the Carlsbad Springs. Here the waters are taken with a strict dietary for three weeks; when the patient is emaciated to a certain degree, he is sent to some other place for recuperation, to return for three successive years. This will often effect a cure. I have a patient who has been benefited by this course. After her return from the first visit to Carlsbad she had three attacks during the year; after the second she had two, and her general health was excellent. She is now returning from her third visit.

Dr. Streets, of the navy, has mentioned to me an interesting case where he used olive oil. The patient, a sailor, was seized with an attack of gall-stones, and was at once put upon the use of olive oil. The discharges were saved, and there were obtained a number of soft, pulpy bodies, which had a very odd appearance. They were somewhat globular, and not faceted. These were placed on a surface so that the oil could be absorbed, and they shrunk in size, showing them to be faceted gall-stones. It seemed as though the oil had penetrated these in some way, either before or after leaving the biliary duct. They were, unquestionably, gall-stones.

Dr. A. B. Hirsh: There is possibly one class of cases in which exception might be taken to Dr. Price's remarks in regard to having a remedy that would obviate future attacks, that is, those cases of distortion of the anatomy of the parts where no operation would avail. In the majority of cases of cholecystotomy we allow for drainage for some time, in the hope that the habit of inspissation of bile may be overcome. Those cases in which peritonitis is associated with resulting obstructive bands, and where, after closure of the fistula the symptoms recur, are not satisfactory ones to deal with. I have met with several such cases, and in these any remedy that offers a promise even of relief should be welcomed.

In regard to the cases reported by practitioners who see but few, and then only at long intervals, there often remains some doubt as to the correctness of their diagnosis. Dr. Porter published in the St. Louis *Weekly Medical Review*, in the Spring of 1889, a paper in which he analyzed a large number of such cases reported of supposed relief of gall-stones by the use of olive oil; he appeared to show that the treatment had no scientific basis; that the cases had not been followed sufficiently long, and that, if anything, the observers had been careless in taking notes.

Dr. Mays: I am here, perhaps, as the innocent champion of olive oil in the treatment of this disease. Having had some very favorable results from its action in three cases of gall-stones, I proposed the subject to the Polyclinic Medical Society for further investigation. From my own experience with it, I think that we ought to be very careful and not make any dogmatic statements concerning the action of this oil. Even if we do not know how an agent acts, we are warranted in ascribing some usefulness to it, if it does the work which was intended it should perform, and especially if we were unable to accomplish this with anything else. I believe the cholagogue action of olive oil has been denied this evening, and in answer to this I would say that Rosenberg's experiments, which show its powerful influence on the biliary secretion, have been before the profession for at least a year and a half. Rutherford found that sodium salicylate was one of the best cholagogues, but the former demonstrated that the olive oil excelled the latter agent as a biliary stimulant. In view of this influence, it is easy to see how it acts in this disease, and it is also easy to see how the sodium salicylate acts beneficially in similar cases.

Dr. Price seems inclined to doubt that the concretions, which are evacuated after the oil is administered, are true gall-stones. This is, of course, difficult to determine in many of the cases which are reported, but at least in one instance (Dr. D. D. Stewart's case) the concretions were examined by Dr. Leffmann, and by him pronounced true gall-stones.

I can hardly agree with the opinion of Dr. Walker that the sending out of inquiries concerning the action of any agent in biliary colic would have brought similar favorable replies. There is too much unanimity, I may say, in the reports favorable to the action of the oil to give the least countenance to such a belief. Besides, we endeavored to guard against this very uncertainty. The circular asked expressly whether any other agents had been given, and with what results. In the great majority of cases all the known remedies had been tried, with doubtful results, or with failures. Many observers stated that no agents had given such signal relief as sweet oil.

If we believe in the efficacy of cholagogues to relieve the attacks of biliary colic, and are in search of an agent having a similar action to prevent their recurrence, then, I think, it is useless to advocate the action of chloroform in this disease, as has been done tonight. So far as I know, chloroform is not a cholagogue, but may act by relieving the spasm of the gall-ducts, and by having a solvent action on the calculi. I can more readily see how olive oil would prevent such recurrence, since it is one of the best stimulants to the hepatic secretion that we possess.

